# ReactionCode: format for reaction searching, analysis, classification, transform, and encoding/decoding

**DOI:** 10.1186/s13321-020-00476-x

**Published:** 2020-12-03

**Authors:** Victorien Delannée, Marc C. Nicklaus

**Affiliations:** Computer-Aided Drug Design Group, Chemical Biology Laboratory, Center for Cancer Research, National Cancer Institute, NIH, 376 Boyles Street, Frederick, MD 21702 USA

**Keywords:** ReactionCode, Reaction, Encoding, Decoding, Searching, Classification

## Abstract

In the past two decades a lot of different formats for molecules and reactions have been created. These formats were mostly developed for the purposes of identifiers, representation, classification, analysis and data exchange. A lot of efforts have been made on molecule formats but only few for reactions where the endeavors have been made mostly by companies leading to proprietary formats. Here, we present ReactionCode: a new open-source format that allows one to encode and decode a reaction into multi-layer machine readable code, which aggregates reactants and products into a condensed graph of reaction (CGR). This format is flexible and can be used in a context of reaction similarity searching and classification. It is also designed for database organization, machine learning applications and as a new transform reaction language.
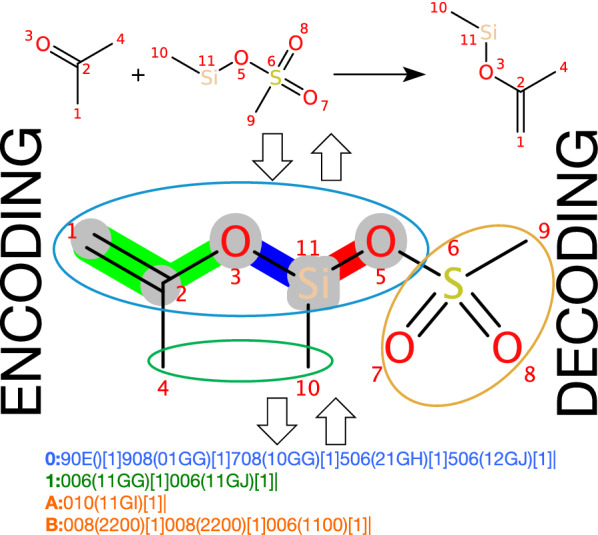

## Introduction

Different proprietary and open formats for reactions have been invented over the past 50 years. The first reaction format can probably be attributed to E. J. Corey and W. T. Wipke. They implemented a format based on rules to generate new molecules and integrated it in the first computer-aided organic synthesis program: OCSS (Organic Chemical Simulation of Synthesis) [1]. This project split to give birth to LHASA (Logic and Heuristics Applied to Synthetic Analysis) [2–4] and SECS (Simulation and Evaluation of Chemical Synthesis) [5]. The LHASA team designed the language CHMTRN (CHeMistryTRaNslator), while the SECS group created the ALCHEM (A Language for CHEMistry) language [6]. After their launch, diverse additional reaction transform languages came up along the implementation of programs such as CLASS and IGOR & IGOR2. However, the arrival of SMILES (Simplified Molecular Input Line Entry System) in the late 1980s led to the development of ReactionSMILES and SMIRKS (SMIles ReaKtion Specification). These two formats were largely adopted by the community and are still widely used nowadays [7–10].

The work around reaction formats has also affected the need for representations and identifiers for data exchange. In the 1990s, Molecular Design Limited (MDL) developed the Chemical Table file (CTfile) format [11]. In this context, the RXNfile and RDfile formats were defined with the objective to store reaction data and quickly became a reference. RXNfile is used to store the structural information for the reactants and products of a single reaction [11], while RDFiles allows one to store a set of RXNs with their associated data [11]. Since then, additional formats have emerged or are under development such as XDfiles [12], MRV [13], UDM [14], CMLReact [15], CDX/CDXML [16] and ReactionSPL [17]. However, none of these formats succeeded in establishing itself widely as the CTfile formats are still much more frequently used. Next to these representations, work on reaction identifiers has also been done. The Reaction International Chemical Identifier (RInChI) [18], an application of InChI [19, 20] was recently developed with the objective to offer a unique reaction identifier, which can help to organize and validate reaction databases [18].

Besides the formats specifically designed to describe reaction transforms and allow easy data exchange, other more versatile formats have been developed in order to try to offer more flexibility and be utilized in different contexts related to reactions. In 1986, Fujita proposed the Imaginary Transition State (ITS) format, which aggregates reactants and products inside a pseudo-molecule in which the bond changes of a reaction are annotated. This pseudo-molecule was created to be used for the purposes of reaction retrieval and design [21]. This format evolved and became known as Condensed Graph of Reaction (CGR). Stored in an SD File, it is mainly employed for machine learning applications, similarity search, and classification [22, 23]. Recently, a SMIRKS-like format for CGR was implemented concomitant with the development of Python-based tools to operate on them (CGRTools) [24]. However, this format cannot be used directly for, e.g., string-based comparisons of reactions. Indeed, all analysis methods using it are based on molecular graph coloration and molecular fragment generated from the CGR [23, 25–27]. Next to the CGR format, three multi-layer formats considering the reaction center and the neighbor atoms have been developed by J. L. Faulon, InfoChem and Elsevier. J. L. Faulon created the reaction signature, where each reactant and product are described as a tree without taking into account the bond type, and calculates the differences between the reactant and product trees [28]. Despite the versatility of this approach, the consideration of only the atom types and their simple connection is a huge limitation. InfoChem developed the reaction ClassCode, which provides a unique identifier (hash) for the reaction center and its two closest atom neighborhood layers [29]. Similarly, Elsevier implemented the BINCODE, which computes, using a pseudo-molecule, a linear string for each layer from the reaction center to the deepest atom neighborhood layers. Each layer contains the atoms that compose it and their connection tables. In addition, the BINCODE also encodes the bond fate and the atom hybridization change [30]. While the ClassCode is limited to a depth of 2 and is strict by its nature as an identifier, the BINCODE appears to offer more flexibility. Indeed, it covers the complete reaction, and its nature as a string allows some modifications for search purposes. However, the BINCODE was made overly generalist by encoding elements into categories (e.g. the halogens Cl, Br, and I have the same encoding). It therefore cannot be used to recover the entire reaction.

To overcome these limitations, we have developed a new format named ReactionCode, which is a multi-layer machine readable code. This open source format is canonical and designed to be flexible, upgradeable and versatile in order to be applied in a broad range of applications. ReactionCode is particularly useful for reaction similarity searching and classification, but is also conceived for machine learning applications and as a new transform reaction language.

**Fig. 1:**
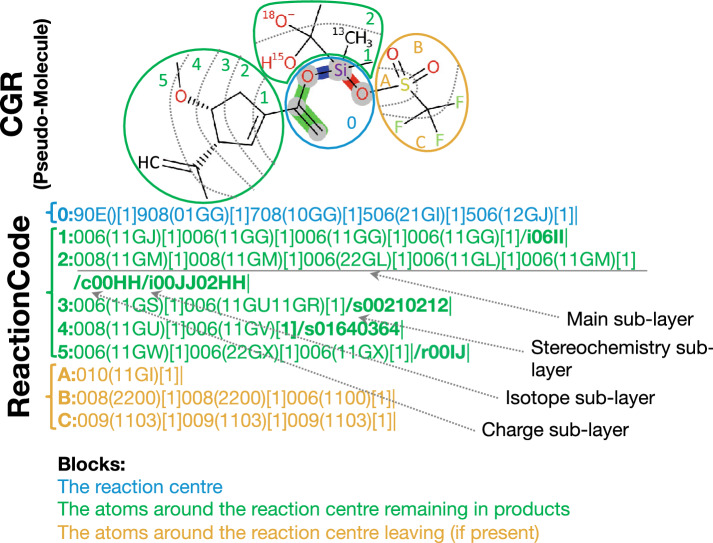
ReactionCode structure: The ReactionCode is composed of three blocks. The first one describes the reaction center (shown in blue) and starts with ‘0:’, which corresponds to depth 0. The entire reaction center (atoms highlighted in gray) is always stored in this single layer. The block in green illustrates the atoms around the reaction center that are still kept in the product(s). This block can be composed of one or multiple layers and each layer starts with a number followed by ‘:’. The figure indicates the depth of the atoms present in this layer in relation to the reaction center. The block shown in yellow encapsulates the atoms around the reaction center which are absent in the product(s). This block can be composed of one or multiple layers and each layer starts with a letter followed by ‘:’. The figure indicates the depth of the atoms present in this layer in relation to the reaction center. (The letter A means a depth equal to one). Each layer is terminated by a ’|’ symbol and is composed of a main sub-layer that starts after the : symbol. The optional layers begin with the ‘/’ symbol. /s characterizes the stereochemistry layer. /c describes the charge layers. /i indicates the isotope layer

**Fig. 2:**
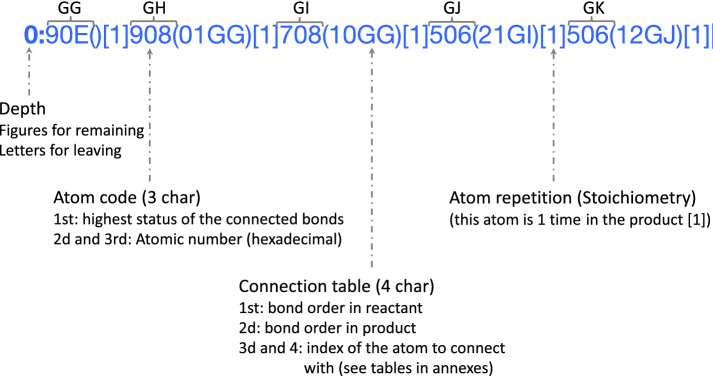
Main sub-layer composition: A layer starts with its depth, which is the distance of its atoms from the reaction center (which has depth = 0). The next three characters characterize the atom. The first character indicates the highest bond status among all bonds connected to this atom. (Additional file 1: Table S7. For instance, the Si atom (90E) is connected by 4 single bonds: 2 are not part of the reaction centre and are encoded by 0; 1 bond is broken, encoded by 7; and another one is made, encoded by 9, which is the highest bond status. The two other characters encode the atom symbol (Additional file 1: Table S8). E.g., 0E stands for Si. The connection table is contained between the brackets. Each bond in the connection table is encoded by 4 characters. E.g., the connection table (11GU11GS) encodes 2 bonds. The first two characters indicate the bond order in reactants for the first one and in products for the second one, respectively (Additional file 1: Table S6). 01GG means a bond is made with the atom at index GG. The last two characters represent the index of the other atom to connect to (see Additional file 1: Table S1 for the reaction center and the remaining group and Additional file 1: Table S2 for the leaving group). The square brackets store the atom stoichiometry, i.e. the number of times a same atom is in the products (Example in Additional file 1: Figure S1)

## Methodology and software

### ReactionCode format

#### Structure

The ReactionCode is a multi-layer machine readable code, which is produced from the aggregation of reactants and products into a condensed graph of reaction (CGR) (Fig. 1). The ReactionCode is organized into three blocks, each of which containing their corresponding layers:

1. Block 1: Reaction center, containing only atoms undergoing changes in bond status (changes in stereochemistry, charge, isotope, or radical status do not qualify an atom as part of the reaction center
2. Block 2: Atoms around the reaction center remaining in the product
3. Block 3: Leaving atoms around the reaction center (if any)

Each layer is composed of a main sub-layer and up to three optional sub-layers, which describes the stereochemistry, the charges, and the isotope, respectively. A layer starts with a number if it illustrates the reaction center or the remaining group, or a letter if it describes the leaving group. It is always terminated by the symbol ‘|’.

*Main sub-layer* The main sub-layer is composed of 4 types of information: the depth, the atom code, the connection table and the atom stoichiometry (Fig. 2). This layer starts with the depth followed by ‘:’. The depth indicates the distance relative to the reaction center. It is expressed in numbers for the reaction center and the remaining group(s) and in letters for the leaving group(s). The atom code is composed of three characters: the first indicates the highest status of the connected bonds encoded using the hexadecimal system (Additional file 1: Table S7), the two others encode the atom type (Additional file 1: Table S8). Each atom code is followed by a parenthesized connection table, which indicates each bond connected to an atom with a lower index. A bond is encoded by 4 characters: the 1st indicates the bond order in reactants, the 2d encodes the bond order in products (Additional file 1: Table S6) and the last two refer to the index of the other atom connected to. The indices are encoded using the hexadecimal system for the atoms to connect that are present in the blocks corresponding to the leaving group (Additional file 1: Table S2) and the indices of atoms in the two other blocks are encoded using a lookup table (Additional file 1: Table S1). Finally, the square brackets store the atom stoichiometry, i.e. the number of times the same atom is in the products (Example in Additional file 1: Figure S1).

**Fig. 3:**
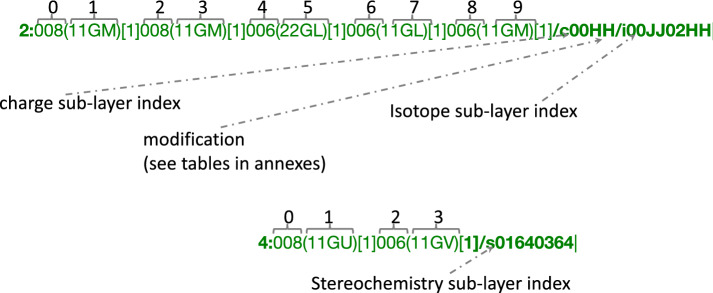
Optional sub-layer composition: An optional sub-layer is directly located after the main sub-layer. Such a code is composed by the symbol ‘/’ followed by a letter and indicates the modification (/c for charge, /i for isotope and /s for stereochemistry). Each block qualifying an atom or a bond is composed of four characters. The first two characters are a number (2 characters) indicating the index (decimal system) of the entity (atom or bond) in the current layer which has to be modified. The next 2 characters are encoding the change to apply to reactants (first character) and products (second character) (for isotope and charge see Additional file 1: Table S3; for atom stereochemistry see Additional file 1: Table S4; and for bond stereochemistry, see Additional file 1: Table S5)

**Fig. 4:**
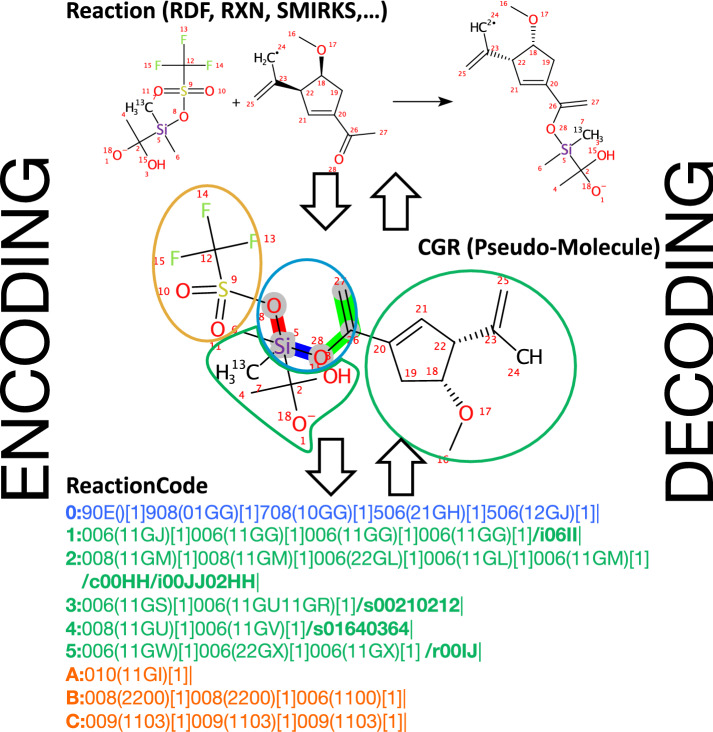
Encoding and decoding process: *Encoding* The reactants and products of the mapped reaction are aggregated into a pseudo-molecule. The bond changes are annotated: green for a bond order change, blue for a bond made, and red for a bond broken. All atoms and bonds are annotated as part of the reaction center (inside blue circle), remaining in the final product (green circles), or leaving the final product (yellow circle). Finally, all atoms and bonds are encoded into ReactionCode by layers starting from the reaction center to the outermost layer. *Decoding:* The ReactionCode is transformed into a pseudo-molecule, which allows one to recover the initial reaction

*Optional sub-layers* The optional sub-layers qualify the atom and bond in their corresponding layer. Only the sub-layer(s) where an atom has a property different from the reference 0 (i.e. has a charge, stereochemistry information, a non-standard isotope, or is a radical) are written directly after the end of the main sub-layer. The priority order is: (1) the charge sub-layer (/c), (2) the stereochemistry sub-layer (/s), (3) the isotope sub-layer (/i), and (4) the radical sub-layer (/r) (Fig. 3).

1. Charge layer: The charge layer starts with /c and the charge information is contained in a block containing the charged atom index (2 digits) and 2 characters encoding the charge. The first one encodes the state in reactants and the second one the state in products (Additional file 1: Table S3). E.g., in /c00HH, “/c” indicates that this layer contains charge information. It having 4 characters means that 1 (4/4) atom has a charge. The only modification is: “00HH”. 00 means that the entity at index 00, which is the atom “008”, is modified. The third character “H” encodes a negative charge $-1$, which remains unchanged in products as the fourth character is encoded by the same letter “H”.
2. Stereochemistry layer: The stereochemistry layer starts with /s and the relative information is contained in a block containing the atom or bond index (2 digits), which has the corresponding stereochemistry modification and 2 characters encoding the stereochemistry in reactants by the first character and in products by the second one (Additional file 1: Tables S4 and S5). e.g., in /s01640364, “/s” indicates that this layer contains stereochemistry information. It having 8 characters means that 2 (8/4) entities [atom(s) and/or bond(s)] have a stereochemistry information. The two modifications are: “0164” and “0346”. The first modification is encoded by the first 4 characters “0164”. 01 means that the entity at index 01, which is the bond “11GV”, is modified. The third character “4” encodes a DOWN bond in reactants, which becomes an UP bond in products indicated by the fourth character “6”. The next 4 characters 0364 modify the bond “11GU” from UP to DOWN.
3. Isotope layer: The isotope layer starts with /i and the isotope information is contained in a block containing the isotope atom index (2 digits) and 2 characters encoding the mass difference between the current isotope and the reference. The first one is for the reactants and the second one for products (Additional file 1: Table S3). e.g., in /i00JJ02HH “/i” indicates that this layer contains isotope information. It having 8 characters means that 2 (8/4) atoms are isotopes. The two modifications are: “00JJ” and “02HH”. 00 means that the entity at index 00, which is the atom “008”, is modified. The third character “J” encodes an addition of 2 neutrons to the common isotope. 008 encodes an oxygen with 2 more neutrons, which means that the atom is an $^{18}$O. The fourth character “H” is unchanged, which indicates that the atoms in products remains the same isotope.
4. Radical layer: The isotope layer starts with /r and the isotope information is contained in a block containing the isotope atom index (2 digits) and 2 characters encoding the mass difference between the current isotope and the reference. The first one is for the reactants and the second one for products (Additional file 1: Table S3). e.g., in /r00IJ “/r” indicates that this layer contains isotope information. It having 4 characters means that 1 (4/4) atom is radical. The modification is “00IJ”. 00 means that the entity is at index 00 in the current sub-layer. The third character “I” encodes an addition of 1 radical (valence equals at 1 for the carbon). The fourth character “J” encodes an addition of 2 radicals (valence equals at 2 for the carbon).

#### Encoding/decoding process

One of the major strengths of ReactionCode is its capacity to be bidirectional: a reaction encoded into ReactionCode can be easily partially or fully decoded to get the reaction back (Fig. 4).

**Fig. 5:**
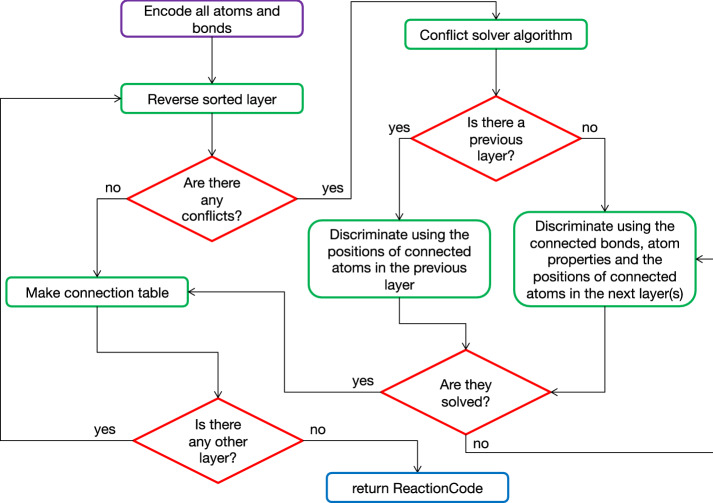
ReactionCode encoding algorithm: Once all atoms and bonds are encoded, all encoded atoms present in the reaction center (depth = 0) are reverse sorted. If two or more atoms have the same code, the conflict solver algorithm is started. The conflict algorithm will make a tree of the connected atom (see Additional file 1: Figure S2) and compare the atom codes and the bond codes of the atom present in the next layer using a BFS algorithm. The algorithm iterates until the conflicts are solved and a proper order can be set up. Then, the connections between the atoms in the current layer are made. Then, all the atoms present in the depth n + 1 (if any), which will be all atoms connected with those in the reaction center in this situation, are reverse sorted. If there are any conflicts, they are first solved by comparing the position of the connected atoms in the previous layer, then using the connected bonds, the atom properties (stereochemistry, charge and isotopy) and finally in the next layer(s) if the conflict cannot be solved. We iterate this process until all layers are processed

**Fig. 6:**
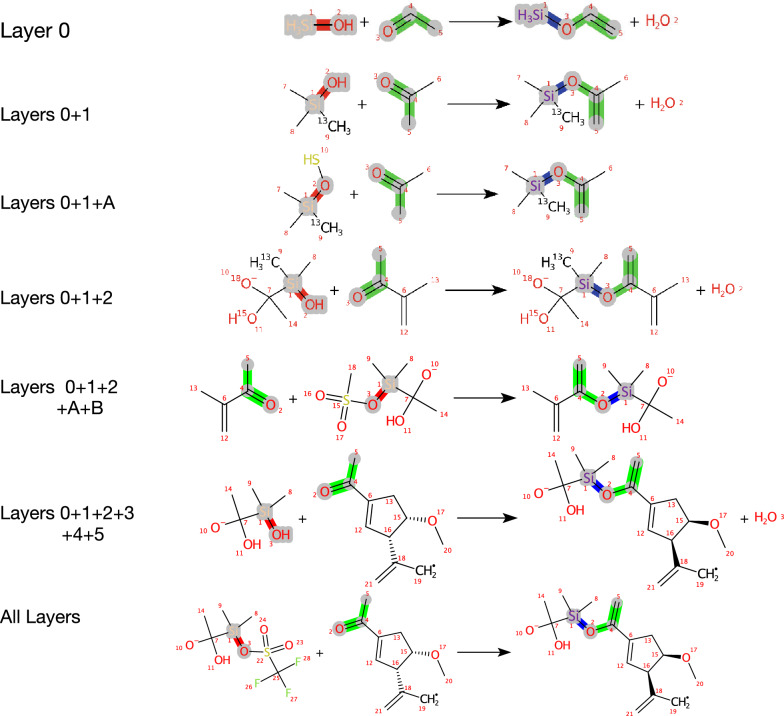
ReactionCode partial and complete decoding: The ReactionCode can be decoded by taking all or some layers. The layer 0 corresponds to the decoding of the reaction center only. Layers 0 + 1 illustrates the decoding of the reaction center and all surrounding atoms in the remaining group having a depth equal to 1, while layers 0 + 1 + A incorporates all surrounding atoms (remaining and leaving groups) with a depth of 1. Layers 0 + 1 + 2 and 0 + 1 + 2 + A + B are considering all surrounding atoms present in the depth lower or equal to 2. Layers 0 + 1 + 2 + 3 + 4 + 5 is an example where the reaction is decoded in its entirety but without the leaving group. Finally, “All Layers” represents the decoding of the complete reaction

In order to generate the ReactionCode, a mapped reaction is necessary. The first step consists in annotating each atom and bond in reactants and products. Three types of annotation are computed:

- atoms and bonds constituting the reaction center
- atoms and bonds present both in reactants and products, which are annotated as the remaining group
- atoms and bonds present in reactants but absent in products (if any), which are annotated as the leaving group

Once the annotation part is finished, reactants and products are aggregated into a CGR. Finally, the ReactionCode is generated from the CGR. Each atom of the CGR is encoded and reverse-ranked by layers. The algorithm starts from the reaction center, reverse-ranks each atom of this layer and makes the connection between them. Then, a Breadth First Search (BFS) algorithm is used to obtain all the surrounding atoms having a depth of 1. These atoms are separated into 2 layers: those belonging to the remaining layer and those that are part of the leaving group. All encoded atoms are reverse-ranked and the connections between each atom with the current and the previous layer are established. The algorithm iterates this procedure until all atoms have been visited (Fig. 5).

The decoding process reconstructs the pseudo-molecule from the ReactionCode by transforming each atom code into an atom object and making the bonds between them. This step relies on the chemoinformatics Java libraries contained in CDK (Chemistry Development Kit) [31]. Then, the pseudo-molecule is transformed into reactants and products in order to get the original reaction back. The ReactionCode is set up by default to recover a balanced reaction but the elements present in the leaving group block could be ignored by the user in order to not have them in the products.

#### ReactionCode software

Java powered by CDK was used to develop the software to generate the ReactionCode, to decode it, to make pseudo-molecules, and to use it as a new transform language. All these functions can be easily used thanks to a CLI (command line interface) and the JAR file can also be directly employed as an API by calling the corresponding class.

*Encoder* The encoder allows one to produce the pseudo-molecules and ReactionCodes. It takes the most common formats as input: SMIRKS (single or a set of SMIRKS in a file), RXN and RDF. The encoder can provide the pseudo-smiles in SDF and in SMILES format and depict them. Finally, the generated ReactionCodes are given in a CSV file.

*Decoder* The decoder allows one to get the original reaction back. The reactions can be provided as reactionSMILES, SMIRKS, RXN, or RDF. They can also depicted as a PNG file. In addition, a partial reaction can be generated by giving the layers of interest as input (Fig. 6).

*Transformer* Thanks to the structure of ReactionCode, where each layer is only dependent on its previous layers but independent of its subsequent layers, it can be used as a transform language where the ReactionCode is transformed into a pattern applied to a set of reactants (Fig. 6). The transformer takes a complete or partial ReactionCode (set of layers) and the reactants as a unique SMILES String or an SD file. If the entire pattern matches the query structures, the transformer will generate all unique possible products. It can output them in reactionSMILES, SMIRKS, RXN, or RDF format.

#### ReactionCode validation

To test the encoding and decoding, we used the UPSTO data set (https://bitbucket.org/dan2097/patent-reaction-extraction/downloads). First, all spectator molecules (i.e. molecules not contributing to the reactions) were removed. Then, all reactions were encoded into ReactionCode. The circular fingerprint ECFP6 was generated for each molecule in both reactions. A reaction was considered similar if all fingerprints of the original reaction were contained in the fingerprints of the decoded reaction. To evaluate the correctness of the reactions returned as not identical, we applied the 3 following protocols:

- Molecules were not kekulized and products were not corrected. All aromatic bonds were set to single and the aromatic property was set to false. The implicit hydrogen was set to 0 for each aromatic atom. All aromatic atoms were set to non-aromatic. We did not apply our algorithm trying to deduce missing cleaved bonds for unbalanced reactions to restore the correct balance by predicting the correct missing product. This procedure allows detecting wrong atom-atom mapping in the USPTO dataset by manually comparing the returned results. The original and the decoded reactions are both depicted and the atom-atom mapping is checked in the original reaction. Removing the aromaticity and not kekulizing the molecules avoid false negatives due to the kekulization, which can produce different tautomers.
- Molecules were kekulized and products were not corrected. All molecules were kekulized. We did not apply our algorithm trying to deduce missing cleaved bonds for unbalanced reactions to restore the correct balance by predicting the correct missing product. This procedure will detect the differences related to the kekulization leading to potentially different tautomers and the failures of our product correction algorithm.
- Molecules were not kekulized and products were corrected. All aromatic bonds were set to single and the aromatic property was set to false. The implicit hydrogen was set to 0 for each aromatic atom. All aromatic atoms were set to non-aromatic. We applied our algorithm trying to deduce missing cleaved bonds for unbalanced reactions to restore the correct balance by predicting the correct missing product. This procedure will detect the differences related to the failures of our product correction algorithm.

The validation procedure code can be found on GitHub (Tests.java).

**Table 1:** ReactionCode validation (tested with version 1.2.0)

Test number	Similar reactions	Non-identical reactions
1	478,948 (99.98%)	87 (0.02%)
2	466,634 (97.41%)	12,425 (2.59%)
		3105 (0.65%) structures could not be kekulized
		9320 (1.94%) structures that are tautomers
3	478,948 (99.61%)	87 (0.39%)

## Applications and results

### ReactionCode validation

The first validation test showed that 87 decoded reactions did not match with the original reactions (Table 1). After manual analysis, we identified the source of these differences coming from an incorrect atom-atom mapping. This result demonstrates the capacity of our software at identifying wrong atom-atom mapping. It also indicated that 99.8% of the dataset is correctly annotated. The second validation test indicated that 12,426 reactions were not identical (Table 1). This count includes 2 types of errors: both reactions are tautomers of each other (9320 reactions) and our kekulization algorithm failed to kekulize the molecule (3105 reactions). The kekulization process does not guarantee the generation of the initial tautomer and can therefore fail for some reactions. Finally, 87 reactions were not well corrected, which were those with a bad atom-atom mapping.

### USPTO reaction data diversity analysis

The USPTO reaction dataset has been used in many machine learning approaches for predicting reactions [32–35]. However, we know of no previous analysis to evaluate the diversity of this dataset. For this purpose, we have used the generated ReactionCodes of each reaction in the USPTO dataset.

To evaluate the diversity, we split the ReactionCodes by incremental layers taking into account a layer and all its previous layers and count the common occurrences. The first part of the analysis consists of extracting all reaction center layers (depth 0) and reverse-sort them as a function of their frequency. In other words, the most frequent reaction center is at the top of the list. Then, the next layers are processed in the same way until we reach a depth of 9, leading to the generation of 10 CSV files (see Additional file 1). Each file starts with the letter ’d’ followed by the depth and contains 2 columns: one with the partial ReactionCode and another one with the number of occurrences (number of time this ReactionCode was found in USPTO dataset).

**Fig. 7:**
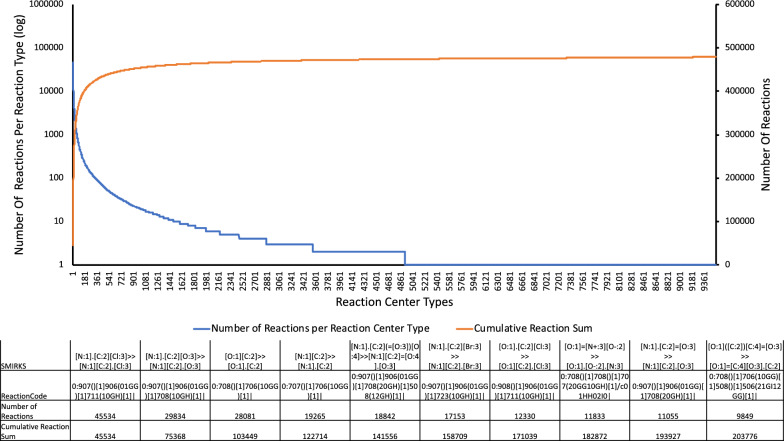
Reaction center diversity analysis: The UPSTO contains 9532 different reaction centers (by extension 9532 reaction types). The graph illustrates the diversity of the reaction types of UPSTO dataset. The x-axis corresponds to the reaction types, where 1 indicates the reaction center, which is the most frequent in the dataset and 9432 the one, which is the less frequent. For instance, the reaction center is common to 45,534 reactions. The blue line shows the number of reaction center types in the logarithmic scale (the reaction center 1 is present in 45,534 reactions, and the reaction center 2 in 29,834 reactions). The orange line depicts the cumulative sum of reactions. For example, the cumulative sum for the reaction center 2 is 75,368 as the reactions center 1 is identified in 45,534 reactions, and the reaction center 2 in 29,834 (45,534 + 29,834 = 75,368). The table details the 10 most frequent reaction centers

**Fig. 8:**
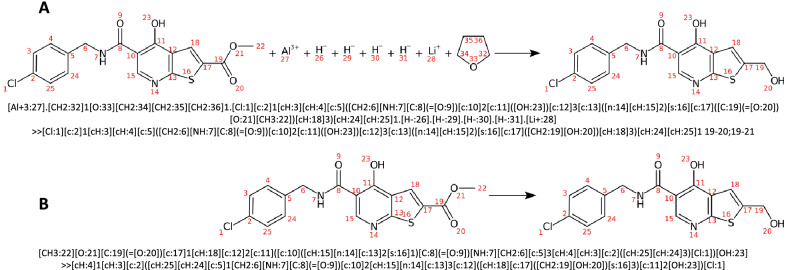
Kekulization differences: Reaction **a** extracted from the USPTO dataset is parsed by CDK and its SMIRKS is recreated after removing the agents (Reaction **b**). The only difference in the regenerated SMIRKS is the atom order. Despite all other characteristics remaining unchanged (atom-atom mapping, aromaticity, implicit hydrogens, etc.), the resulting SMIRKS leads to a different kekulization (kekulization of the ring containing atoms 10–15 in the reactants)

**Fig. 9:**
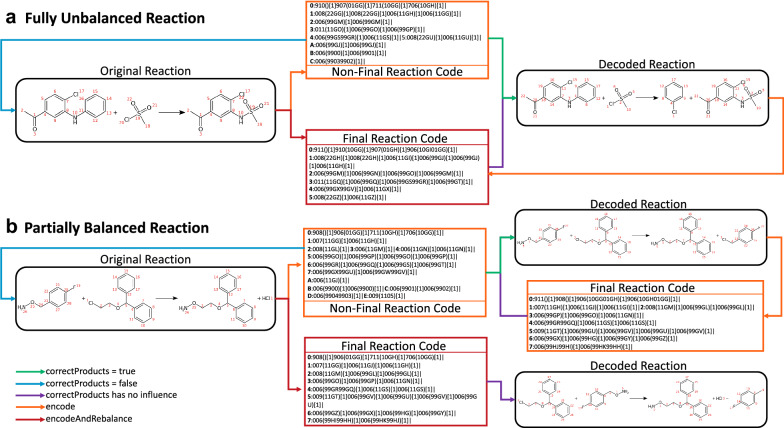
Reaction balancing and product correction: The function encodeAndRebalance attempts to correct the missing products in the original reaction by deducing missing to-be-generated bonds. The encode function generates a non-final ReactionCode, which is still able to reproduce the original unbalanced reaction. If the parameter correctProducts is set to true for the decoding, the products related to the missing atom in the given reaction are corrected. Reaction**a** depicts a fully unbalanced reaction. As there is no reference in the products, the correction algorithm returns the same decoded reaction using the non-final or final ReactionCode. Reaction **b** depicts a partially balanced reaction ((i.e.unbalanced reaction with more than one products)), where one atom involved in a broken bond is present. The function encodeAndRebalance returns a different reaction because it flags the atoms in products, which do not need to be corrected ([Cl:3]). While decoding the non-final ReactionCode, this information is missing and as the Cl atom is not connected to any atom in the remaining layers or leaving layer, it is considered by our algorithm as leaving and is associated with the other leaving group

The USPTO is formed of 479,035 reactions. Among these reactions, 9532 different reaction centers were identified by ReactionCode, i.e. our approach determines that the UPSTO dataset contains 9532 reaction types. The 10 most-represented reaction types in this dataset are found in 203,776 (42.5%) of the reactions. 90% of the USPTO dataset is covered by only 400 reaction types, which corresponds to 4.2% of all reaction types identified in this dataset (Fig. 7. We finally note that 4607 reaction types (48.3%) are only represented by one single reaction in the USPTO dataset.

### Other applications of ReactionCode

ReactionCode is a format that can be used for multiple purposes. We describe a few of them here.

### Reaction balancing correction

Unbalanced reactions (typically, one or more molecules are missing in products) are not uncommon in reaction databases. This can complicate or entirely throw off analyses and work-up of reactions. As ReactionCode aggregates both reactants and products, it can be used to restore the balance of a reaction by encoding and then re-decoding the flawed reaction.

### Searching for similar reactions

The ReactionCode is perfectly suited to search for similar reactions in a database as it is in string format. In addition, a wild card can replace each figure or letter. This can be employed in order to match with any atom, or to ignore the bond order, or any property desired by the user. The syntax of the ReactionCode thus provides the user with a broad flexibility.

### Reaction transform language

The ReactionCode is also designed as a new reaction transform language. One or multiple layers can be used to match a set of reactants in order to generate all the possible products and get all possible reactions. This can be easily done by using our software. Note, however, that this approach does not incorporate any knowledge about the actual synthetic accessibility of the proposed reaction (in contrast to CHMTRN/PATRAN [36]) but operates strictly on the basis of pattern matching.

### Classification

The layered structure of the ReactionCode allows one to classify the reaction in order to make statistical analyses, study the diversity or just to have an idea of the contents of a database. A clusterization of reaction data can also be useful in the context of machine learning, for trying to build the best possible training, testing, and validation sets.

### Machine Learning

The ReactionCode could be useful for machine learning applications as descriptors or directly for reaction prediction by predicting one or multiple layers. The ReactionCode describes the reaction center and its neighboring environment, which provides additional descriptors compared to current methods.

### Compression

In the context of graph databases, the ReactionCode could be used as a tree structure where a node corresponds to a layer. This structure could improve the searching process but also help save disk usage because only the unique layers are stored. This structure permits one to retrieve and regenerate each reaction. Such a tree structure could be used to develop a reaction encoding process. Each layer could be transformed into a bit vector similarly to fingerprints used for molecules, which could allow one to compress a reaction and speed up the reaction comparison process.

## Discussion

### ReactionCode validation

Kekulization leads to an increase in the number of non-similar reactions. The original kekulization algorithm in CDK first identifies the atoms that can receive a pi bond. Then, it attempts to find a perfect match, such that a pi bond is located next to each atom being able to have a pi bond, and it then propagates the bond order information. If the solution is ambiguous, the kekulization is aborted. Such ambiguity is often related to missing or ambiguous implicit hydrogen(s) or a failure of the aromaticity perception algorithm. As ReactionCode does not store implicit hydrogen information, we implemented an algorithm to force kekulization only for final reactions (decoded reactions, which are not used for transforming). This can generate some errors. To potentially fix them in a future version, the implicit hydrogen number for each atom could be encoded in ReactionCode, or some manual patterns for kekulization correction could be implemented. However, it may not be guaranteed to reproduce the right Kekulé structure for both cases.

In general, dealing with kekulized molecules can potentially be a source of mistakes. First, some errors can be made by the algorithms during the aromaticity perception. Second, each toolkit has a different kekulization algorithm, which can lead to potentially different kekulized molecules. Thus, different tautomers can be the cause of the non-application of a transform as ReactionCode will lose aromaticity information and look instead for a specific bond layout. Third, the generation of different Kekulé structures is a common problem as two different SMIRKS of the same reaction can lead to different kekulized molecules. For instance, Fig. 8 shows the same reaction, which is depicted differently by https://www.simolecule.com/cdkdepict/depict.html. The reaction A is the raw reaction found in the USPTO dataset. The reaction B is this same reaction but it has been previously parsed by CDK and its SMIRKS has been recreated after removing the spectator molecules. To avoid such errors related to kekulization, and merge different kekulized molecules, ReactionCode uses the aromaticity instead of the bond order. However, the bond order can be encoded in the API instead of the aromaticity information by setting all isAromatic() properties for atoms and bonds to false beforehand.

All atoms and bonds present in the leaving layers correspond to the atoms and bonds absent in the products in the submitted reaction. By extension, the presence of leaving layers implies an unbalanced reaction. As ReactionCode contains information on both reactants and products, it can restore the balance while decoding. However, for some reactions, bond(s) made between two non-hydrogen atoms can be missing, which would lead to the generation of incorrect products. To attempt to fix these products, we have implemented an algorithm that tries to deduce the missing to-be-generated bonds by using the information stored in the leaving layer A and the atom in the reaction center having 7 as its status. A bond between two atoms is generated if one of them is a carbon, the other one is a non-heteroatom and both are not detected in the original products.

ReactionCode implements a dedicated function encodeAndRebalance, which encodes the reaction having a leaving group and flags the leaving atoms present in the given product. Then, the generated ReactionCode is decoded using our correction algorithm by taking into account the flagged atoms. Finally, the fixed reaction is re-encoded. The created ReactionCode is final (i.e. the decoding/re-encoding will always give the same ReactionCode) as the atoms and bonds contained in the leaving group have been repositioned in the remaining group. The user can still encode and decode the reaction twice, but an unbalanced reaction with a reaction partially balanced (i.e.unbalanced reactions with more than one products), can potentially lead to a wrong leaving product (Fig. 9b). This experimental algorithm trying to correct the unbalanced reactions can still be improved. For instance, it could be ameliorated by using reaction patterns validated by chemists to correct the products. As this implementation is still experimental, the API can prevent its usage by setting the parameter correctProducts to false. If correctProducts is set to false, the leaving group will be removed from the products and the unbalanced reaction would be regenerated (Fig. 9).

### USPTO analysis

The diversity analysis of the USPTO dataset showed that this database is covered in the vast majority by only about 400 reaction types while conversely 48.3% of the dataset consists of reactions that do not share a common reaction center with any other reaction in the dataset. This analysis shows that the USPTO has an unbalanced diversity with some significantly over-represented reaction types, which may explain the good accuracy of the models predicting reactions. However, as 48.3% of the dataset consists of unique reactions, it may be wise to define an appropriate strategy during training, testing and the validation of a predictive model. The unique reactions cannot be learned by ML (if they are in the validation dataset it will decrease the score, if they are in the training set, they cannot be validated). In other words, if one does not apply cross-validation, you cannot trust such models. We therefore hope that this diversity analysis of the USPTO dataset via ReactionCodes may be helpful for better sampling during model-building and for future implementations using the USPTO dataset.

### Future developement

The ReactionCode was designed to be an upgradeable format. This format is open to the community, which can submit a new version. For instance, the aromatic bonds are encoded with the same character “9”, which can fail to encode some tautomeric reactions. In a lot of mapped reactions, the correspondence of the Kekulé versions in reactants and products is wrong, which will be considered as a change of the molecule and integrated into the reaction center. To avoid this problem, it is safest in most of the cases to adopt the aromatic annotation. However, if the user is sure of his/her mapping, this parameter can be easily changed and the bond will be encoded as single or double. Besides, it can be of interest for tautomeric reaction studies to have the number of hydrogens in the ReactionCode. This can be easily added to the ReactionCode code by modifying one single parameter in the code.

## Conclusion

ReactionCode has been implemented as a new, open source, versatile reaction format that avoids the drawbacks of others. The field of its possible applications is large and we believe that it can be profitable for the community working on reactions. Freely available and open source software has been developed to generate the ReactionCode from, and to convert it to, a variety of existing reaction formats, as well as to use it as a reaction transform language. This program and the source code are available at https://cactus.nci.nih.gov/reactioncode.

## Supplementary information

**Additional file 1: Figure S1**. Stoichiometry management. **Figure S2**. Conflict solver algorithm. **Table S1**. Atom index encoding for reaction center and remaining group. **Table S2**. Atom index encoding for leaving group. **Table S3**. Charge and Isotope encoding. **Table S4**. Atom stereochemistry encoding. **Table S5**. Bond stereochemistry encoding. **Table S6**. Bond order encoding. **Table S7**. Bond change status encoding. **Table S8**. Atom symbol encoding.

## Data Availability

The software is available at https://cactus.nci.nih.gov/reactioncode and the source code at https://github.com/victoriendelannee/reactioncode. Future updates will be available at the same URLs. The generated data for USPTO diversity analysis is attached to the manuscript as 11 additional files.
