# Supplementary material for: ReactionCode: format for reaction searching, analysis, classification, transform, and encoding/decoding
Source: J Cheminform. 2020 Dec 3;12:72. doi: 10.1186/s13321-020-00476-x (PMC7713369; doi:10.1186/s13321-020-00476-x)
Supplement: Supplementary file 1 — Additional file 1: Figure S1. Stoichiometry management. Figure S2. Conflict solver algorithm. Table S1. Atom index encoding for reaction center and remaining group. Table S2. Atom index encoding for leaving group. Table S3. Charge and Isotope encoding. Table S4. Atom stereochemistry encoding. Table S5. Bond stereochemistry encoding. Table S6. Bond order encoding. Table S7. Bond change status encoding. Table S8. Atom symbol encoding. [file 13321_2020_476_MOESM1_ESM.pdf]

figStoichiometry.pdf

Figure S1 Stoichiometry management The green molecule has 2 Br atoms, which both react with the amine function of the blue molecule. The stoichiometry is 2 blue molecules for and 1 green molecule. Consequently, each atom of the green molecule has to be represented 2 times in the product, which is encoded by [2].

figConflictSolver.pdf

Figure S2 Conflict solver algorithm After ranking each atom according to their codes, a conflict algorithm solves the conflicted atoms (i.e. those sharing the same code). This algorithm discriminates first by looking at atoms in the previous layers. If the conflict can not be solved, the bond type is used. If the two previous processes have failed, the priorities of the connected atoms in the next layer(s) are analyzed. **A:** The carbon atom with an orange atom code (006) is ranked before the carbon with a purple atom code (006). The orange carbon has a higher priority because its connected atom (Si atom with atom code 70E) in the previous layer is ranked first, while the purple carbon atom is connected to the atom at position 2 (O atom with atom code 708). **B:** As the first method failed to solve the conflict, the bonds are taken into account. This takes into account the encoded bond types and the number of connected bonds with both layers. All bond codes related to the connected bonds of a conflicted atom are reverse sorted and merged into a string. The merged code for the carbon atom with an atom code in orange is 2211 and the one for the carbon atom with an atom code in purple is 111111. The carbon atom with an atom code in orange is ranked first because the priority of its merged code is higher ($2211 > 111111$) by using the string comparison method: compareTo). **C:** As both previous methods failed to solve the conflict, the atom priorities are analyzed in the current layer. In this example, the two carbons (906) were ranked by looking at the next layers. The carbon 906 in bold and black is positioned 2nd because its connected Br atom (with an atom code in orange) has a higher score than the Br with the purple atom code connected to a different carbon (906). **D:** If the current layer method cannot discriminate the conflicts, the atom priorities are analyzed in the next layer. The oxygen atom with an atom code in orange has a higher priority than the one with an atom code in purple. The orange oxygen is connected to a nitrogen (007) atom, which has a higher priority than the carbon connected to the oxygen with purple atom code ($007 > 006$)). **E:** In this example, the algorithm iterates over the next layers until being able to solve the conflict. The carbon atom with an atom code in orange has a higher priority than the one in purple. The orange atom has an oxygen atom in layer n+2, while the purple atom has a carbon atom in the same layer. Oxygen has a higher rank than carbon ($008 > 006$). In the case of symmetry, where both paths are equivalent, a unique position is attributed to each atom and is used as a reference for solving the next conflicts (i.e. sub-figure C, where both 906 carbons are ranked and used to discriminate the ranking of both bromines (723)).

Additional Files

Figure S1 — Stoichiometry management

Figure S2 — Conflict solver algorithm

Table S1 — Atom index encoding for reaction center and remaining group
Table S2 — Atom index encoding for leaving group
TableS3 — Charge and Isotope encoding
Table S4 — Atom stereochemistry encoding
Table S5 — Bond stereochemistry encoding
Table S6 — Bond order encoding
Table S7 — Bond change status encoding
Table S8 — Atom symbol encoding

	G	H	I	J	K	L	M	N	O	P	Q	R	S	T	U	V	W	X	Y	Z
G	1	2	3	4	5	6	7	8	9	10	11	12	13	14	15	16	17	18	19	20
H	21	22	23	24	25	26	27	28	29	30	31	32	33	34	35	36	37	38	39	40
I	41	42	43	44	45	46	47	48	49	50	51	52	53	54	55	56	57	58	59	60
J	61	62	63	64	65	66	67	68	69	70	71	72	73	74	75	76	77	78	79	80
K	81	82	83	84	85	86	87	88	89	90	91	92	93	94	95	96	97	98	99	100
L	101	102	103	104	105	106	107	108	109	110	111	112	113	114	115	116	117	118	119	120
M	121	122	123	124	125	126	127	128	129	130	131	132	133	134	135	136	137	138	139	140
N	141	142	143	144	145	146	147	148	149	150	151	152	153	154	155	156	157	158	159	160
O	161	162	163	164	165	166	167	168	169	170	171	172	173	174	175	176	177	178	179	180
P	181	182	183	184	185	186	187	188	189	190	191	192	193	194	195	196	197	198	199	200
Q	201	202	203	204	205	206	207	208	209	210	211	212	213	214	215	216	217	218	219	220
R	221	222	223	224	225	226	227	228	229	230	231	232	233	234	235	236	237	238	239	240
S	241	242	243	244	245	246	247	248	249	250	251	252	253	254	255	256	257	258	259	260
T	261	262	263	264	265	266	267	268	269	270	271	272	273	274	275	276	277	278	279	280
U	281	282	283	284	285	286	287	288	289	290	291	292	293	294	295	296	297	298	299	300
V	301	302	303	304	305	306	307	308	309	310	311	312	313	314	315	316	317	318	319	320
W	321	322	323	324	325	326	327	328	329	330	331	332	333	334	335	336	337	338	339	340
X	341	342	343	344	345	346	347	348	349	350	351	352	353	354	355	356	357	358	359	360
Y	361	362	363	364	365	366	367	368	369	370	371	372	373	374	375	376	377	378	379	380
Z	381	382	383	384	385	386	387	388	389	390	391	392	393	394	395	396	397	398	399	400

Table S1 Atom index encoding for reaction center and remaining group

	0	1	2	3	4	5	6	7	8	9	a	b	c	d	e	f
0	0	1	2	3	4	5	6	7	8	9	10	11	12	13	14	15
1	16	17	18	19	20	21	22	23	24	25	26	27	28	29	30	31
2	32	33	34	35	36	37	38	39	40	41	42	43	44	45	46	47
3	48	49	50	51	52	53	54	55	56	57	58	59	60	61	62	63
4	64	65	66	67	68	69	70	71	72	73	74	75	76	77	78	79
5	80	81	82	83	84	85	86	87	88	89	90	91	92	93	94	95
6	96	97	98	99	100	101	102	103	104	105	106	107	108	109	110	111
7	112	113	114	115	116	117	118	119	120	121	122	123	124	125	126	127
8	128	129	130	131	132	133	134	135	136	137	138	139	140	141	142	143
9	144	145	146	147	148	149	150	151	152	153	154	155	156	157	158	159
A	160	161	162	163	164	165	166	167	168	169	170	171	172	173	174	175
B	176	177	178	179	180	181	182	183	184	185	186	187	188	189	190	191
C	192	193	194	195	196	197	198	199	200	201	202	203	204	205	206	207
D	208	209	210	211	212	213	214	215	216	217	218	219	220	221	222	223
E	224	225	226	227	228	229	230	231	232	233	234	235	236	237	238	239
F	240	241	242	243	244	245	246	247	248	249	250	251	252	253	254	255

Table S2 Atom index encoding for leaving groups

C or i	encoding		C or i	encoding
1	I		-1	H
2	J		-2	G
3	K		-3	F
4	L		-4	E
5	M		-5	D
6	N		-6	C
7	O		-7	B
8	P		-8	A
9	Q		-9	9
10	R		-10	8
11	S		-11	7
12	T		-12	6
13	U		-13	5
14	V		-14	4
15	W		-15	3
16	X		-16	2
17	Y		-17	1

Table S3 Charge and Isotope encoding

Symbol	Type	Shorthand	Numeric shorthand	encoding
@TH1	Tetrahedral	ANTI CLOCKWISE (=LEFT)	1	1
@TH2	Tetrahedral	CLOCKWISE (=RIGHT)	2	2
@AL1	ExtendedTetrahedral	ANTI CLOCKWISE (=LEFT)	1	3
@AL2	ExtendedTetrahedral	CLOCKWISE (=RIGHT)	2	4
@DB1	DoubleBond	OPPOSITE	1	5
@DB2	DoubleBond	TOGETHER	2	6
@CT1	ExtendedCisTrans	OPPOSITE	1	7
@CT2	ExtendedCisTrans	TOGETHER	2	8
@SP1	SquarePlanar		1	9
@SP2	SquarePlanar		2	A
@SP3	SquarePlanar		3	B
@TB1	TrigonalBipyramidal	ANTI CLOCKWISE (=LEFT)	1	C
@TB2	TrigonalBipyramidal	CLOCKWISE (=RIGHT)	2	D
@OH1	Octahedral	ANTI CLOCKWISE (=LEFT)	1	E
@OH2	Octahedral	CLOCKWISE (=RIGHT)	2	F
@AP1	Atropisomeric	ANTI CLOCKWISE (=LEFT)	1	G
@AP2	Atropisomeric	CLOCKWISE (=RIGHT)	2	H

Table S4 Atom stereochemistry encoding

Type	encoding
E	1
Z	2
DOWN INVERTED	3
DOWN	4
UP INVERTED	5
UP	6
E or Z	7
UP or DOWN	8
UP or DOWN INVERTED	9

Table S5 Bond stereochemistry encoding

1	SINGLE
2	DOUBLE
3	TRIPLE
4	QUADRUPLE
5	QUINTUPLE
6	SEXTUPLE
9	AROMATIC
0	NONE

Table S6 Bond order encoding

score	meaning	encoding
0	unmarked	0
-1	not a reaction centre	0
1	a reaction centre	1
4	bond order changes	4
6	bond broken	6
8	bond made	8
5	4 + 1 (is a center and order changes)	5
7	6 + 1 (is a center and bond broken)	7
9	8 + 1 (is a center and bond made)	9
10	6 + 4 (bond is both broken and its order changes)	A
11	10 + 1 (is a center, bond broken and order changes)	B
12	8 + 4 (bond is both made and its order changes)	C
13	12 + 1 (is a center, bond made and order changes)	D

Table S7 Bond change status encoding

4, 6, 8 are always encoded to 5, 7, 9, respectively
-1 is increased to 0
10 is reduced to 4
11 is reduced to 5
12 is reduced to 8
13 is reduced to 9
Note: The current code version (1.2.0) only uses scores 5, 7 and 9.

H=1																	He=2
Li=3	Be=4											B=5	C=6	N=07	O=08	F=09	Ne=0A
Na=0B	Mg=0C											Al=0D	Si=0E	P=0F	S=10	Cl=11	Ar=12
K=13	Ca=14	Sc=15	Ti=16	V=17	Cr=18	Mn=19	Fe=1A	Co=1B	Ni=1C	Cu=1D	Zn=1E	Ga=1F	Ge=20	As=21	Se=22	Br=23	Kr=24
Rb=25	Sr=26	Y=27	Zr=28	Nb=29	Mo=2A	Tc=2B	Ru=2C	Rh=2D	Pd=2E	Ag=2F	Cd=30	In=31	Sn=32	Sb=33	Te=34	I=35	Xe=36
Cs=37	Ba=39	La=39	Hf=48	Ta=49	W=4A	Re=4B	Os=4C	Ir=4D	Pt=4E	Au=4F	Hg=50	Tl=51	Pb=52	Bi=53	Po=54	At=55	Rn=56
Fr=57	Ra=58	Ac=59	Rf=68	Db=69	Sg=6A	Bh=6B	Hs=6C	Mt=6D	Ds=6E	Rg=6F	Cn=70	Nh=71	Fl=72	Mc=73	Lv=74	Ts=75	Og=76

La=39	Ce=3A	Pr=3B	Nd=3C	Pm=3D	Sm=3E	Eu=3F	Gd=40	Tb=41	Dy=42	Ho=43	Er=44	Tm=45	Yb=46	Lu=47
Ac=g9	Th=5A	Pa=5B	U=5C	Np=5D	Pu=5E	Am=5F	Cm=60	Bk=61	Cf=62	Es=63	Fm=64	Md=65	No=66	Lr=67

R=FF	*=FE	^{2}H=FD	^{3}H=FC

Table S8 Atom symbol encoding

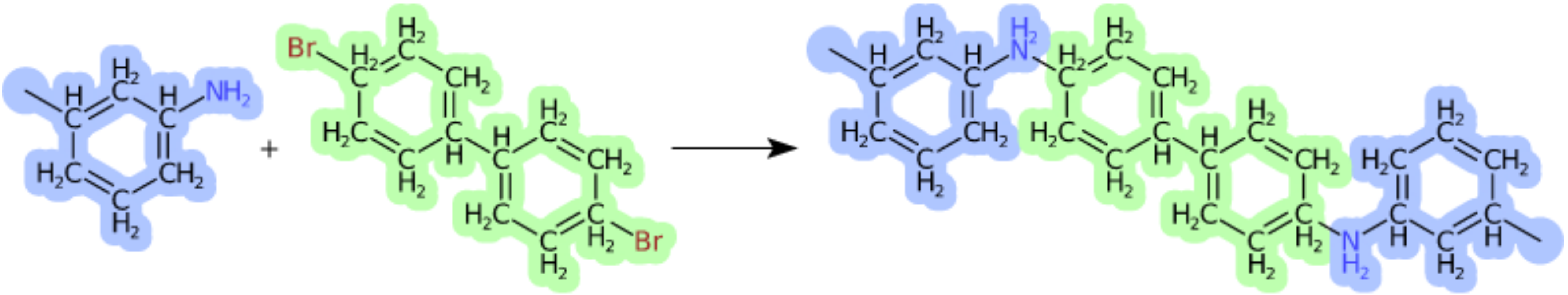

0:907()[2]906(01GG)[1]906(01GG)[1]723(10GH)[1]723(10GI)[1]|
1:006(11GG)[2]006(99GH)[1]006(99GH)[1]006(99GI)[1]006(99GI)[1]|
2:006(99GN)[1]006(99GP)[1]006(99GL)[2]006(99GL)[2]006(99GM)[1]006(99GO)[1]|
3:006(99GU99GQ)[1]006(99GW99GV99GR)[1]006(99GT)[2]006(99GS)[2]|
4:006(99GZ99GY)[2]006(11GY)[2]|

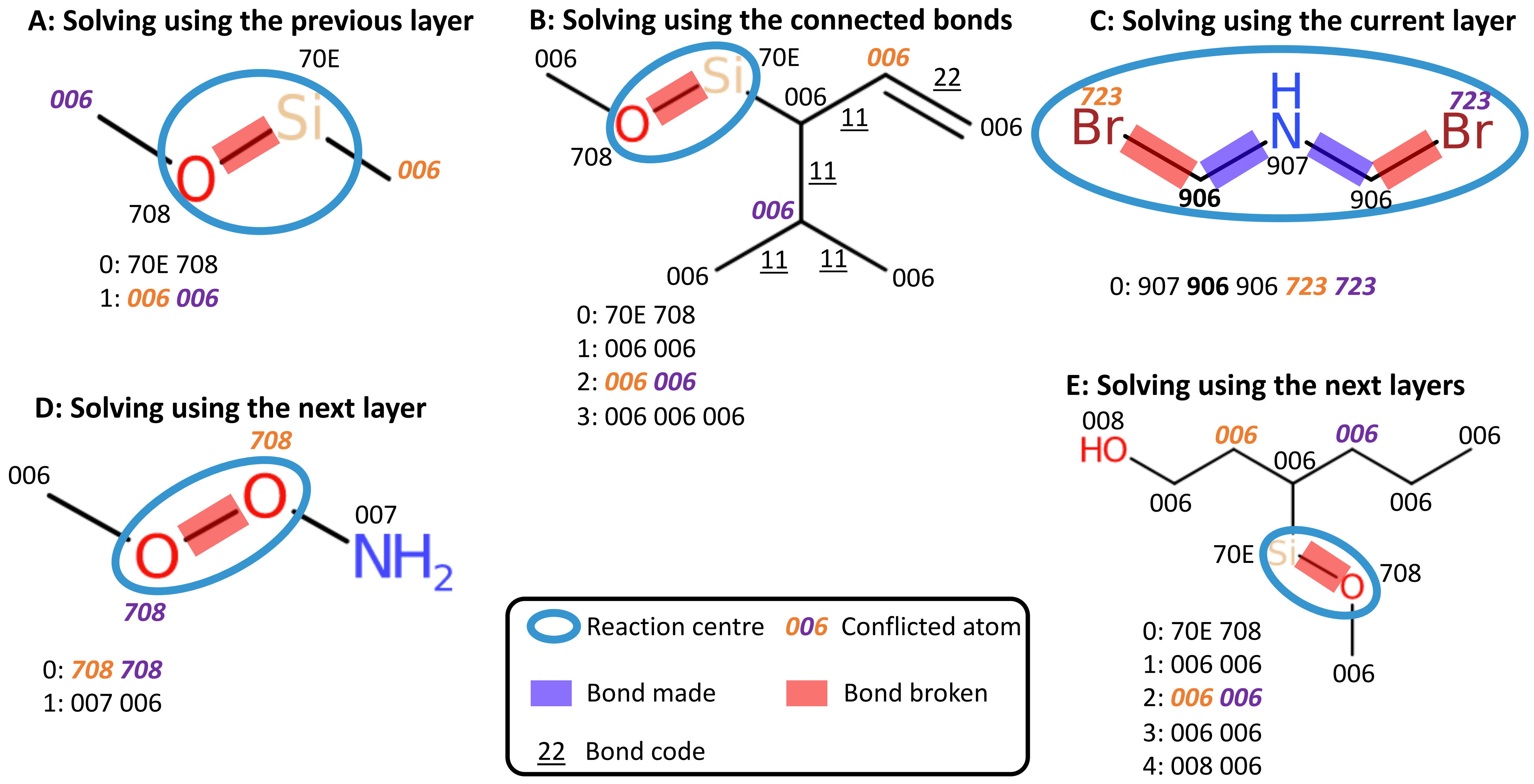
A: Solving using the previous layer
70E
006
Si
O
006
708
0: 70E 708
1: 006 006
B: Solving using the connected bonds
006
Si
70E
006
22
006
11
O
006
708
11
006
11
11
006
006
0: 70E 708
1: 006 006
2: 006 006
3: 006 006 006
C: Solving using the current layer
723
H
723
Br
N
Br
907
906
906
0: 907 906 906 723 723
D: Solving using the next layer
708
006
O
007
O
NH2
708
0: 708 708
1: 007 006
E: Solving using the next layers
008
006
006
006
HO
006
006
006
70E
Si
O
708
006
0: 70E 708
1: 006 006
2: 006 006
3: 006 006
4: 008 006
Reaction centre
006 Conflicted atom
Bond made
Bond broken
22 Bond code
